# Exploring visual short‐term memory binding tests as early markers of cognitive decline: A comparative study between healthy aging and mild cognitive impairment

**DOI:** 10.1002/alz.092306

**Published:** 2025-01-03

**Authors:** Monika McAtarsney‐Kovacs, Shahina Pardhan, Raju Sapkota, Ian van der Linde

**Affiliations:** ^1^ Anglia Ruskin University, Cambridge United Kingdom

## Abstract

**Background:**

Elderly people, especially those with mild cognitive impairment (MCI), are at heightened risk of developing dementia. In this study, we examined how well healthy young, normally aging and MCI participants performed in visual short‐term memory (VSTM) binding, a cognitive process in which an item’s different visual properties (e.g., form, location, colour) are combined and stored momentarily in our mind before being forgotten. Earlier work has identified VSTM binding as a potential cognitive skill that may be disproportionately affected in those with MCI/dementia, meaning that it may serve as an early behavioral marker.

**Method:**

Three groups of participants were recruited: 34 healthy young adults (mean age 25±3 years), 52 normally aging older adults (mean age 71±6.4 years) and 16 older adults with MCI (mean age 74±6years). All participants completed computer‐based VSTM tasks requiring the binding of object‐location, object name‐location, colour‐location, and colour word–location for two or four sequentially presented stimuli (presentation duration <1 sec). Participants also completed standard cognitive tests (Addenbrookes Cognitive Examination, Montreal Cognitive Assessment, Trail Making Test, National Adult Reading Test and CANTAB Paired Associates Learning). We compared the performance of healthy young and normally aging older groups to examine the effect of aging; normally aging older and MCI groups were compared to examine the effect of MCI.

**Result:**

The performance of the normally aging older group was significantly lower than that of the healthy young group for memory tasks requiring object‐location (p = .003) and colour‐location (p = .014) binding. The performance of the MCI group was significantly lower than that of the normally aging older group for object name‐location (p = .014), colour‐location (p = .036) and colour word‐location (p<.001) VSTM tasks. VSTM binding performance correlated well with standard cognitive tests (ps ≤ .05).

**Conclusion:**

The performance of the young healthy group exceeded that of both older groups. Not surprisingly the MCI group performed least well, particularly in tasks assessing object name‐location, colour‐location and colour word‐location binding. The disproportionate deterioration of these abilities implies that they may serve as early markers of the cognitive decline associated with MCI/dementia, and may form the basis of straightforward cognitive tests that may complement standard cognitive tests.

